# Modulation of Host Immune Response Is an Alternative Strategy to Combat SARS-CoV-2 Pathogenesis

**DOI:** 10.3389/fimmu.2021.660632

**Published:** 2021-07-08

**Authors:** Lakhveer Singh, Sakshi Bajaj, Manoj Gadewar, Nitin Verma, Mohd Nazam Ansari, Abdulaziz S. Saeedan, Gaurav Kaithwas, Manjari Singh

**Affiliations:** ^1^ School of Medical and Allied Sciences, KR Mangalam University, Gurgaon, India; ^2^ Chaudhary Devi Lal College of Pharmacy, Yamuna Nagar, India; ^3^ Department of Pharmacology and Toxicology, College of Pharmacy, Prince Sattam Bin Abdulaziz University, Al-Kharj, Saudi Arabia; ^4^ Department of Pharmaceutical Sciences, Babasaheb Bhimrao Ambedkar University, Lucknow, India; ^5^ Department of Pharmaceutical Sciences, Assam Central University, Silchar, India

**Keywords:** SARS-CoV-2, type-2 pneumocyte, ACE-2 receptor, TMPRSS2, cytokines, macrophages

## Abstract

The novel SARS-CoV-2virus that caused the disease COVID-19 is currently a pandemic worldwide. The virus requires an alveolar type-2 pneumocyte in the host to initiate its life cycle. The viral S1 spike protein helps in the attachment of the virus on toACE-2 receptors present on type-2 pneumocytes, and the S2 spike protein helps in the fusion of the viral membrane with the host membrane. Fusion of the SARS-CoV-2virus and host membrane is followed by entry of viral RNA into the host cells which is directly translated into the replicase-transcriptase complex (RTC) following viral RNA and structural protein syntheses. As the virus replicates within type-2 pneumocytes, the host immune system is activated and alveolar macrophages start secreting cytokines and chemokines, acting as an inflammatory mediator, and chemotactic neutrophils, monocytes, natural NK cells, and CD8+ T cells initiate the local phagocytosis of infected cells. It is not the virus that kills COVID-19 patients; instead, the aberrant host immune response kills them. Modifying the response from the host immune system could reduce the high mortality due to SARS-CoV-2 infection. The present study examines the viral life cycle intype-2 pneumocytes and resultant host immune response along with possible therapeutic targets.

## Introduction

TheCOVID-19 outbreak due to a novel viral disease has variable symptoms which include fever, cough, headache, fatigue, difficulties in breathing, and loss of taste and smell. The symptoms arise from 1-14 days after exposure to the virus. The symptoms may change from mild to moderate and critical in some conditions. Around 15% of people develop severe symptoms like hypoxia and lung damage and5% of people suffer from critical symptoms like respiratory failure, shock, and multiorgan failure (1). The disease slowly progresses to acute respiratory distress syndrome (ARDS) following a lung injury if appropriate measures are not taken promptly. Initially, the infection was recognized due to the severe acute respiratory syndrome coronavirus(SARS-CoV-1)virus that caused an epidemic in 2002 (2). However, when currently available antiviral drugs failed and a high degree of human-to-human transmission was observed, it was realized that it was a new virus outbreak.

The first case was reported in the Wuhan city of China in December 2019.In the next few days, several patients with the same symptoms were admitted to hospitals, and all those patients had a direct or indirect link with the Huanan seafood market of Wuhan, Hubei province (3). Since, most of the patients had a direct or indirect link with the sea food market, it was suggested that the likely source of new virus might have been an animal (4). The virus spreads from human to human through the air from droplets (cough or sneeze), close personal contact (touching), objects or surfaces with viral particles, or from fecal and urine contamination (5). Due to its high transmission rate, the virus covered the whole world, and WHO had to declare coronavirus disease (COVID-19) as a public health emergency (6). Since its origin in Wuhan, more than 28 million people have been infected and more than 0.9 million people have lost their lives, and the number is continuously increasing. At the end of January 2021, the number of global deaths was at 2 million and the number of cumulative cases was about 100 million (John Hopkins, Coronavirus Resource Center) (7). Maximum death shave been reported in Italy, Spain, Iran, the UK, and US compared to Wuhan, the origin of the virus. Firstly, it was observed that the virus belonged to the large family of beta coronaviruses causing respiratory illness but based on its phylogenetic analysis, it was found that it had only an 80%sequence similarity with the deadly SARS-CoV-1(that caused an epidemic in 2002) virus and only a 50%sequence similarity with Middle East respiratory syndrome (MERS, that caused another outbreak in 2012) (8). None of the Coronaviridae family of viruses showed an identical sequence similarity with the new coronavirus. Therefore, the virus is a novel coronavirus discovered in 2019and its disease was formerly designated as COVID-19. Since it has 80% sequence similarity with the previously knownSARS-CoV-1 virus, it is called the SARS-CoV-2 virus and as the first patient was reported in Wuhan, the virus is also called Wuhan human virus (9).

After confirmation of the novelty of the virus, the next question was, what was its origin? SARS and MERS viruses had bats as the common origin but had different intermediary hosts to infect human beings (10). Based on this information, the SARS-CoV-2virus was assumed to have originated and mutated in bats and used an unidentified intermediary host to cause infection in humans (11).

However, due to its novelty, we currently have minimal vaccine therapy to combatSARS-CoV-2 infection. There is no such specific and effective treatment available for this disease, the only current option is management. The patient can be cured by supportive care which includes treatment to relieve symptoms, fluid therapy, oxygen support, and medications to support the affected vital organs (12). In mild cases, supportive care includes medication like paracetamol to relieve symptoms like fever and body aches. The US Center for Disease Control and Prevention (CDC) recommends that those who are suspected to have the disease can isolate at homes and always use face masks and gloves. People with more severe symptoms require treatment in hospitals. Those with low oxygen levels need oxygen support and dexamethasone as it reduces the risk of death. For breathing difficulties, ventilation support and admission to an intensive care unit (ICU) is recommended (13). Several antiviral and antimalarial drugs have been studied in clinical trials and recommended in emergency use in hospitals like hydroxychloroquine, ritonavir, and remdesivir. These drugs are not recommended for early treatment (14).

Nowadays, various vaccines are available for COVID-19 and after taking the vaccines, some countries are free of this disease. A list of vaccines is listed in **Table 1**. Along with that Defence Research and Development Organisation (DRDO) India issued a drug named 2-deoxy-D-glucose (2DG) which will be used as an anti-COVID drug in India.

**Table 1:** Approved vaccines for COVID-19 disease.

S.No.	Name of vaccine	Approved Country	Manufactured Company	Efficacy
1	Moderna	United States	ModernaTX, Inc.	Approximately 92%
2	Pfizer–BioNTech	Multinational	Pfizer Inc.	Approximately 95.3%
3	Sputnik Light	Russia	Gamaleya Research Institute of Epidemiology and Microbiology	Approximately 79.4%
4	Sputnik V	Russia	Gamaleya Research Institute of Epidemiology and Microbiology	Approximately 91%
5	Oxford–AstraZeneca	Multinational, United States	Oxford University and AstraZeneca	Approximately 81.3%
6	BBIBP-CorV	Multinational	Sinopharm	Approximately 93%
7	CoronaVac	Brazil, Turkey	Sinovac Biotech	Approximately 84%
8	Novavax	United Kingdom, South Africa	Novavax and the Coalition for Epidemic Preparedness Innovations (CEPI)	Approximately 89.3%
9	Johnson & Johnson	Multinational, United States, Brazil, South Africa	Janssen Pharmaceuticals Companies of Johnson & Johnson	Approximately 66.3%
10	Covaxin	India	Bharat Biotech	Approximately 78%
11	Convidecia	Multinational	CanSino Biologics	Approximately 65.7%
12	Covidshield	India	Central Drug Standard Control Organization (CDSCO)	Approximately 70%

As far as immune response is concerned, a unique immune response has been observed in COVID-19 patients. Recently, it was observed in a study that in normal patients, there was a lower level of classical inflammatory cytokines like G-CSF, CCL-20, IL-1β, IL-2, IL-6, IL-15, TNF-α, and TGF-β while at the same time, there was a higher plasma level of GM-CSF and CXCL-10 in COVID-19 patients (15). All the observed cytokines are important components of the innate immune response. Some scientists carried out meta-transcriptomic sequencing to analyze the innate immune cells in bronchoalveolar lavage fluid of eight COVID-19 cases. The study reported the overexpression of pro-inflammatory genes, those of chemokines along with innate immune cells like dendritic cells and macrophages. These studies indicated robust activation of the innate immune response in SARS-CoV-2-infected patients. While most of the studies have mainly focused on the innate immune response, little is discussed about the adaptive immune response (16). Although, several studies have reported about the life cycle and heightened immune response raised by the human body against SARS-CoV-2 infection, none of the works could explain the exact mechanism. The results of various studies on the immune system are ambiguous.

The current review is an effort to unveil the viral structure, life cycle, and molecular sites that could be targeted to fight COVID-19 infection. The study also deals with the host immune response raised against SARS-CoV-2 infection and its modulation to minimize alveolar damage.

## Structural Characteristics of SARS-CoV-2

Coronaviruses are spherical in shape (diameter125 nm). A club-shaped protrusion of spike glycoprotein emanates from the lipid bilayer and looks like a crown hence the name “corona” viruses. It belongs to the β-genera of the four identified categories of coronavirus (including α, β, γ, δ) (17). Among all the categories, αand βCoVs are found to infect mammals, γ-coronaviruses infect avian species, and δ-coronaviruses infect both mammals and birds (18). Other proteins embedded on the lipid bilayer are the envelope protein and membrane protein, also present on the virus’s surface, but their structure and function are not well known (19). The genome of the virus is comprised of positive-sense single-stranded RNA that remains encapsulated in the lipid bilayer along with capsid protein. Although little is known about the viral structure, recent work has helped considerably to reveal the virus’s microstructure (20). Daniel Wrapp and colleagues studied the structure of the spike protein using electron microscopy. They stated that the spike protein comprises three subunits (i.e., is trimeric) arranged in such a way that it helps in virus attachment with the angiotensin-converting enzyme (ACE-2) receptor (21).The study also reported that the trimeric proteins of SARS-CoV-2have a 10-20% higher binding affinity with the human ACE-2 receptor compared toSARS-CoV-1 (21, 22).

Further, they explained that this spike protein exists in a metastable perfusion conformation that undergoes substantial structural rearrangement to fuse with the host membrane (21) (23). It is reported that some cross-reactivity of monoclonal antibodies raised against the SARS-CoV structural proteins with SARS-CoV-2 was produced by SARS-CoV-1againstSARS-CoV-2 (24). t is also reported that the spike protein has a furin cleavage site at the boundary of S1 and S2, which is processed before it binds with the host receptor (25). The cross-reactivity of SARS-CoV-1 antibodies against the SARS-CoV-2 virus is also reported (26). This furin-type cleavage was not reported in the SARS-CoV-1 virus. From this, one can speculate why the virus has a high rate of transmission (27). Numerous studies have reported a glycan shield over the spike protein that might be somehow helping in the immunity evasion of the virus, but further work is still needed o confirm its role in immunity evasion (28, 29). There is much more that needs to be discovered about this spike protein. Once we have understood the molecular structure and spatial orientation of the protein residues of the spike protein, we can design its inhibitors accordingly (30).

The genetic material of SARS-CoV-2 is comprised of a large positive-sense single-stranded RNA that remains underneath with a nucleocapsid, which can be directly translated into viral structural and non-structural proteins and the genome of progeny virus (31). The whole SARS-CoV-2genome consists of 29,891 nucleotides, which encodes 9860 amino acids, and these amino acids are used to make 27 types of proteins needed by the virus. Although SARS-CoV-2shares approximately 80% sequence similarity with SARS-CoV-1, there is a significant difference of 380 new amino acids. These new amino acids could be responsible for the extra proteins found in the SARS-CoV-2 virus. For instance, the 8a protein present in SARS-CoV-1isabsent in SARS-CoV-2, and the 84 amino acid protein 8b in SARS-CoV-1 has 121 amino acid residues in SARS-CoV-2 (32). The difference of these 380 amino acids might have contributed to the production of these proteins and the high rate of transmission and immune escape.

Although little is known about these 27 viral proteins encoded by SARS-CoV-2 RNA, scientists now knows about the four major proteins encoded by the virus, which make up the major structure of the virus surface (33). These include spike surface glycoprotein (already discussed above), the small envelope protein, the matrix protein, and the nucleocapsid protein (18).

The spike glycoprotein of the newly emerged SARS-CoV-2 contains a potential cleavage site for furin proteases (34). This observation has implications for the zoonotic origin of the virus and its epidemic spread. The membrane of coronaviruses harbors a trimeric trans-membrane spike (S) glycoprotein, which is essential for the entry of virus particles into the cell (17). The S protein contains two functional domains: a receptor-binding domain and a second domain which contains sequences that mediate fusion of the viral and cell membranes. The S glycoprotein must be cleaved off by the host cell proteases to enable its fusion with the host ACE receptors. The S glycoprotein is necessary for viral fusion and entry into the host cells; therefore, it is also on the list to prepare a vaccine against SARS-CoV-2 (35).

Structural and molecular understanding helps researchers to correlate the innate and adaptive immune response mechanisms against SARS-CoV-2. Here, we aim to discuss the molecular involvement of alveolar pneumocytes and the innate and adaptive immune responses toSARS-COV-2infection.

## Pathophysiology of Alveolar Pneumocytes

The wall of alveoli contains type-1 and type-2 pneumocytes that play an important role in the exchange of gases. Type-1 pneumocytes are responsible for gaseous exchange while type-2 pneumocytes secrete a surfactant that keeps the lungs’ surface tension minimal, thus preventing them from collapsing. Alveolar epithelium is in close proximity with blood capillaries that have the thickness of one cell, again required for an efficient exchange of gases during the phenomenon of breathing (36). As alveolar tissue has direct access to external air, normal physiological function of pneumocytes alters in various pathological conditions like pneumonia, tuberculosis, chronic obstructive pulmonary disease (COPD), acute respiratory distress syndrome (ARDS), and asthma. Reduced production of surfactant in cigarette smokers has been attributed in the pathogenesis of COPD. Cigarette smoking stimulates production of reactive oxygen species (ROS) which damage type-2 pneumocytes. Damaged type-2 pneumocytes are phagocytosed by the macrophages and neutrophils that further release inflammatory cytokines and ultimately lead to the inflammation of airways which finally develops into COPD (37). Pneumonia is another pathological condition characterized by an inflamed airway and pus-filled air sacs caused by bacterial, viral, and fungal infection of pneumocytes. Inhaled bacteria injure alveolar cells which further releases leukotriene B4 (LTB4) that acts in a chemotactic manner for the blood cells (monocytes and neutrophils) which rush towards the site of injury. Macrophages in response to engulfed bacteria release interleukin-1(IL-1), IL-8, and tumor necrosis factor-α (TNF-α) which not only raise body temperature but also actin a chemotactic manner from immune cells. Due to the hyper response of secreted cytokines, fluid accumulates in and around the alveoli as consolidation. Consequently, gaseous exchange becomes extremely difficult and the patient struggles to breathe (7). ARDS is a pathological condition resulting from acute lung injury which is associated with various etiologies. Inflammation in ARDS further leads to diffuse alveolar injury, pulmonary edema, infiltration of immune cells, and formation of a hyaline membrane. Like pneumonia, acute lung injury in ARDS also triggers the innate immune response involving macrophages, neutrophils, cytokines, and denudation of the basement membrane of the lungs followed by accumulation of fluid in the alveoli and subsequently respiratory failure (38). Interestingly, the same types of pathological changes have also been observed in patients suffering from COVID-19which has been discussed in depth in the preceding section.

## Multivariate Role of ACE-2 in COVID-19

It has been reported that the presence of ACE-2 is necessary and sufficient to initiate COVID-19 infection (39). It provides a gateway for COVID-19 entry into cells and subsequently infection (17). Therefore, it becomes necessary to study the ACE-2 enzyme and its location on all body cells. ACE-2 is a transmembrane protein of the renin angiotensin system (RAS) family of proteins. It exerts its biological action by converting soluble plasma protein angiotensinogen into angiotensin-I and angiotensin-II which acts on angiotensin type-1 receptors (AT1Rs) and angiotensin type-2receptors (AT2Rs) (39). Although significantly less information is available about their pathophysiological roles, findings from numerous studies have suggested their protective role in the heart, kidney, blood vessels, and central nervous system (40). Scientific research suggests that the ACE-2 receptor protects the alveolar epithelium tissue present in the lung alveoli, consisting of a single layer of epithelial tissue, and enhances proliferation of type-2 pneumocytes in the case of lung fibrosis (41). Another study suggests that well-differentiated cells are the target of COVID-19 as it is easier to enter and egress across the plasma membrane of these cells (42). Alveolar type-2 pneumocytes serve both these purposes. A secondary complication of the virus infection might be due to the high distribution ofACE-2 receptors on the arteries, heart, kidneys, intestine, and lungs (43).

## Viral Life Cycle in Pneumocytes

**Figure 1:**
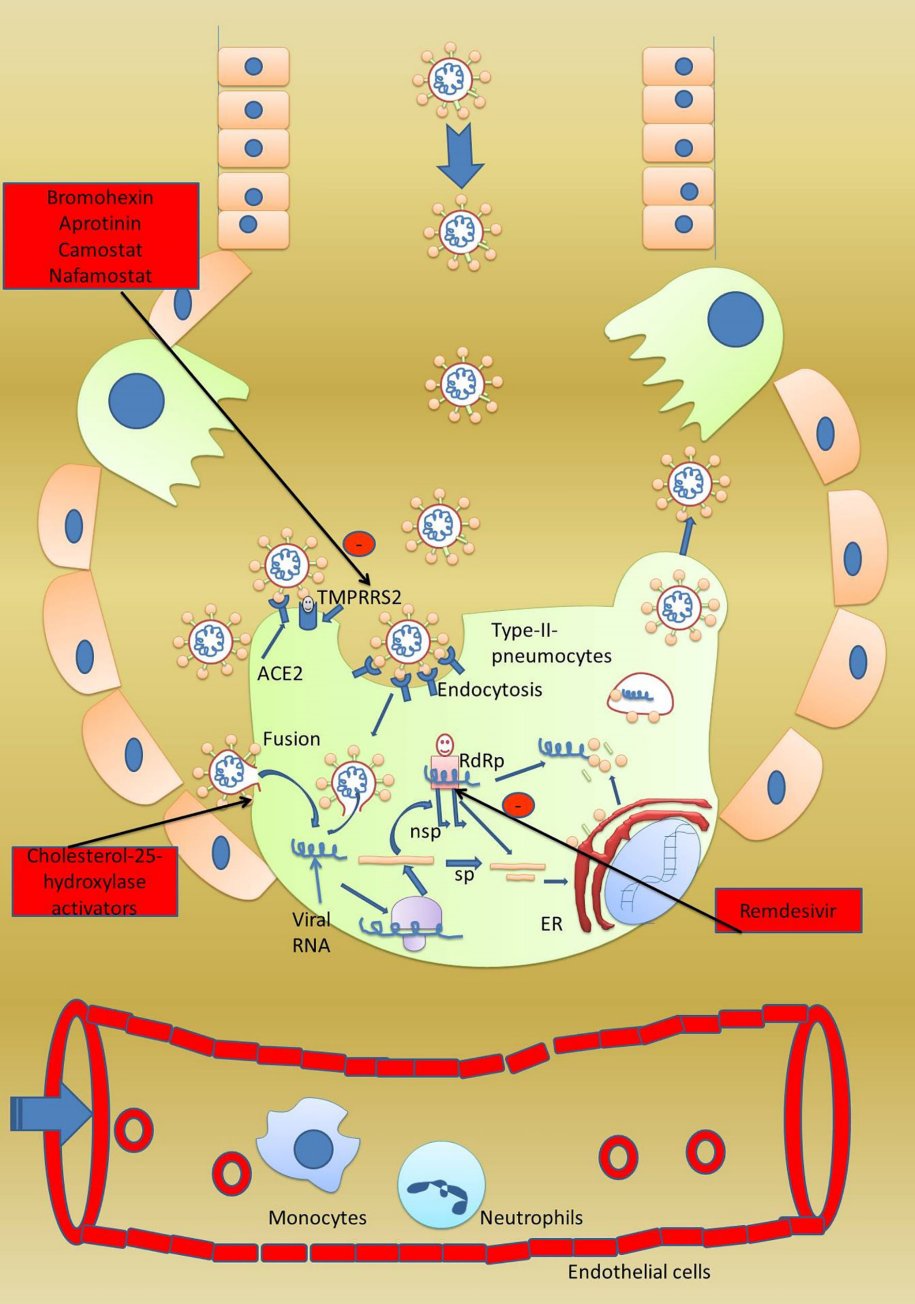
Viral life cycle in the host cell. A healthy person gets infected when they come in contact with an infected person. Once at the surface of type-2 pneumocytes, virus has two options to gain entry into the pneumocytes. First, the virus can directly enter the pneumocytes by a fusion process and secondly by receptor-mediated endocytosis. For receptor-mediated endocytosis, attachment with the ACE-2 receptor is a must. Host serine protease TMPRSS2 activates the spike protein and subunits S1 and S2 get separated. S1 binds to the host ACE-2 receptor while S2 initiates the fusion of the viral envelope with the host plasma membrane followed by formation of viral syncytium and entry into host cells. Once the virus has reached inside the cell, the viral genome is directly translated into major proteins which are further fragmented into smaller proteins which are used in the synthesis of progeny virus. The fragmented proteins are further processed in the endoplasmic reticulum and Golgi body to assemble into progeny virus. Host RNA-dependent RNA polymerase converts the viral RNA into new virions genome. Progeny virus after completing the life cycle bursts the host cells and starts infection into nearby cells. In severely infected patients, the virus also affects the vital organs like kidneys and heart. Potential drug targets 1. Drugs enhancing stabilization of the spike protein can prevent viral entry, 2. inhibitors of TMPRSS2 can stop activation of viral protein, 3. antibodies against S1 and S2 can prevent attachment with host ACE-2, and 4. a fusion inhibitor can prevent viral entry.

The life cycle of COVID-19 is straight-forward. Once it reaches the lungs, it binds to type-2 pneumocytes of the alveoli using its spike protein (36). At the site of the target cells, S proteins are activated by serine protease TMPRSS2, which opens the trimeric proteins into S1 and S2 proteins (20) (**Figure 1**). Another study confirmed that TMPRSS2 and ADAM17 intracellular proteases also helped ACE-2enable the efficient entry of the virus (44). Heurich et al. reported that co-expression of ACE-2 with TMPRSS2 augmented SARS-S uptake. It was reported in the study that arginine residue at 697 and 716 is essential for cleaving of TMPRSS2 serine proteases that mediated the viral S-protein entry (45). The collective effect of TMPRSS2/HAT and ADAM17 on activation of the S protein on COVID-19 and the ACE-2 receptor on type-2 pneumocytes again might be attributable to the high rate of transmission of COVID-19 from person to person. S1 binds with its C-terminal on the ACE-2 receptor present on the pneumocytes which helps in its attachment. Once a firm ACE-2 attachment is fixed, the S2 subunit comes forward to initiate the fusion with the host cells’ plasma membrane and injects its positive-sense single-stranded RNA into the host cells (40). Firstly, in the pneumocytes, the host cells’ ribosome is hacked to translate the viral double-positive single-stranded RNA (++dsRNA) into a large polypeptide which is further chopped into smaller structural and non-structural proteins. The RNA-dependent RNA polymerase (RdRp)is one such non-structural protein that replaces the viral genome (46). Remdesivir, as the inhibitor of RdRp, is currently being used in some cases to combat viral infection (47). The viral genome replicated by RdRp and translated structural proteins like the envelope (E), membrane (M),and spike (S) proteins are then assembled into new virions in the Golgi body. The new virus egresses out of the cell by exocytosis (48).

## Host Immune Response to SARS-CoV-2

### Innate Immune Response

Our nonspecific immunity produces a rapid response to viral infection and acts as the first line of defense. This allows our body time to prepare the specific immunity that needs to be triggered. It is always there in our body and responds as soon as a pathogen invades our body (49). Innate immunity involves physical and chemical barriers and includes cellular response. Major cell types involved in this are leukocytes, neutrophils, monocytes, eosinophils, basophils, and natural killer cells (50). While, neutrophils and monocytes carry out phagocytosis of the invaded pathogen to impart the innate cellular response, natural killer cells kill the invaded pathogen by inducing apoptosis. Apart from this, the complement system also plays a vital role in innate immunity (51).

**Figure 2:**
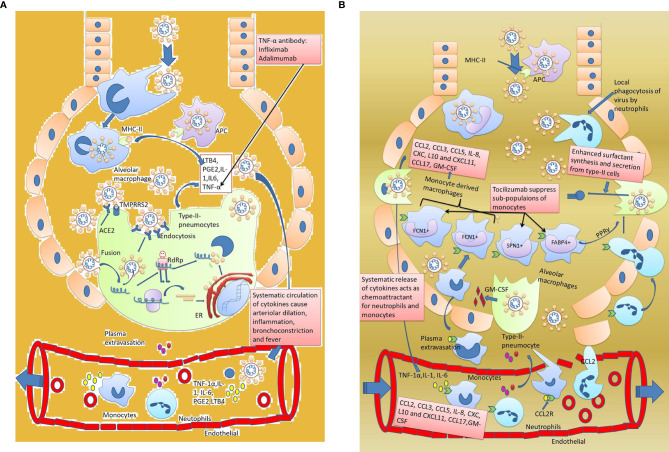
Innate immune response against SARS-CoV-2. **(A)** Early innate immune response and development of symptoms. Host immune response activatestype-2 pneumocytes invaded by the virus. Inflammatory cytokines IL-1, IL-6, and TNF-1α activate the macrophages. Macrophages in acute cases release small proportions of inflammatory markers that not only attract the circulatory monocytes and neutrophils at the site but also increase the body temperature. **(B)** Delayed innate immune response and development of severe pneumonia. Circulating inflammatory mediators IL-1, IL-6, and TNF-α in blood further exacerbate the acute innate immune response by increasing the chemotactic effect of monocytes and neutrophils towards the lungs. The monocytes and neutrophils leak out through the endothelial cells and enter into the alveoli. Neutrophils starts phagocyting the viral particles and monocytes subdivide into macrophages (like FCN1+ macrophages, SPN+1 macrophages, and FABP4+ macrophages). Out of the four sub divisions of macrophages, FCN1+ macrophages are highly inflammatory and produce cytokines like CCL-2, CCL-3, CCL5, IL-8, CXL, L10, CXCL11, CCL17, and GM-CSF which appear as a cytokine storm in SARS-CoV-2. The second type of macrophages, i.e., SPN+ macrophages, have an anti-inflammatory effect while FABP+ macrophages enhance surfactant secretion by type-2 pneumocytes through PPARγ expression. As a result, excess fluid accumulates in the alveoli resulting in severe pneumonia.

Innate immune response quickly responds to the SARS-CoV-2virus whenever a healthy person comes in contact with an infected person (**Figure 2A** **)**. The initial innate response is presented by the mucus and cilia of the trachea, which induces coughing and excess mucus to help promote the virus’ early expulsion. Various studies have reported these symptoms in COVID-19 patients (52, 53). But viruses that escape from the mucus and cilial lining of the trachea finally reach the alveoli and enter type-2 pneumocytes (37). As the virus replicates in these cells, patients remain asymptomatic up to two weeks post-infection. But continuous virus replication causes a cellular injury that involves the synthesis and secretion of inflammatory mediators like leukotrienes, prostaglandin, and histamine, which make up the second line of the innate response (54, 55). The main goal of inflammation is to isolate, destroy, and inactivate the invader, remove debris, and repair the injured tissue. Due to inflammation, circulating neutrophils and monocytes rush to the site of infection, where they start phagocytosis of the invading virus (15).

Further, these cells also secrete IL-1, IL-8, and TNF-α,enhancing the chemotactic effect of the circulating monocyte and neutrophil cells to squeeze through blood capillaries to reach the extravascular region and finally the alveoli (56). Apart from pneumocytes, the virus also infects macrophages present in the lung’s alveoli and the infection of type-2 pneumocytes on their surface (antigen presenting cells) to activate the adaptive immune response (37). Macrophages also secrete IL-1 and TNF-α, which act on the hypothalamus which causes high fever and acts upon the bone marrow, which causes leukocytosis (56). Virally infected cells also produce a lot of interferons (IFNs) which play an essential role in innate (IFN-α and β) and adaptive immune responses (IFN-γ) (57).

### Fate of Macrophage- and Neutrophil-Mediated Cytokine Storm

Phagocytosis is followed by the formation of pseudopodia, the engulfment of the virus and the formation of the phagosome, and then a fusion of lysosome-containing vesicles which causes hydrolysis and the degradation of the ingested virus antigens.

In neutrophils, the viral antigens are finally released into the extracellular region from where they reach the lymph nodes and activate B; cells in the adaptive immune system. Along with that, if the engulfed virus is too strong to be degraded into antigens, then the neutrophils undergo self-lysis through a free radical-induced mechanism (58). Oxygen in neutrophils is transformed into free radicals like O2, H2O2, and HOCl, which cause lysis of the viral cell but also cause damage to the neutrophil’s nucleus (59). There is one more mechanism known as neutrophil extracellular traps (NETS) by which neutrophils can inhibit viral replication. The nucleic material released from degraded neutrophils moves to the extracellular region, binds to the antigen with its histone protein, and causes lysis of that antigen. Then, cathepsin-like enzymes initiate hydrolysis of the complex thus formed (60, 61).

Monocytes have a different fate after phagocytosis. The hydrolyzing released antigens are not released into the circulation but are thoroughly processed inside the monocytes and expressed on the major histocompatibility complex-I and II. MHC-I is expressed by all nucleated cells, but MHC-II is only presented by the antigen-presenting cells (macrophages, dendritic cells, and B-lymphocytes) (62). The monocyte-derived macrophages reach the lymph nodes, which also stimulate an adaptive immune response.

The complement system is a significant player in the immune system. It further enhances microbial clearing by phagocytic cells utilizing liver complement proteins and antibodies (63). The complement system involves the activation of a cascade of reactions with the attachment of antibodies on the antigen to form a membrane attack complex (MAC), which includes a channel in the antigen cells. Water and ions leak out from the cells, and ultimately lysis of antigenic cells takes place (63). There are three pathways through which the complement system can act:

1. **Classical pathway:** The pathway is initiated to bind antibodies to the antigen present on the foreign cell. C1 is the first complement protein that attaches to the Fc part of the antibody. Subsequently, complement proteins C4, C2, C3b, C5b, C6, C7, C8, and C9 bind to each other and form a long complex. The complex breaks at the interface of C3band C5b. The pentameric complex comprises an MAC that leads to the lysis of cells. The remaining complex acts as opsonin for the circulating macrophages and is phagocytized. Released complement C3a and C5a are acted upon by the protease released by mast cells, which are activated and augment the inflammatory response by attracting monocytes and neutrophils and further augment the inflammatory response (64).
2. **Alternative pathway:** The complement protein directly binds (without an antibody) with an antigen present on the foreign cell and initiates a cascade reaction that involves subsequent attachment with C5b, C6, C7, C8, and C9. Finally, the complex breaks at the interface of C3b and C5b, and the rest of the steps are like the classical pathway (65).
3. **Lectin pathway:** The pathway starts with the binding of lectin with mannose molecules present on the antigen. Attachment of lectin is followed by complement proteins C4, C2, C3b, C5b, C6, C7, C8, and C9. The rest of the steps are like the classical pathway (66).

### Differential Crosstalk of Cytokine Blockade

Apart from the circulating complement proteins, virally infected cells produce interferons released into the extracellular region and bind with the receptors on healthy cells and stimulate them to produce degrading enzymes. When these healthy cells get infected with the same virus, the enzymes activate and kill the invading virus. The enzymes break the viral messenger RNA and thus viral protein synthesis (67). Further, IFNs enhance the phagocytic activity of macrophages, stimulate the production of antibodies by βcells, and enhance the killing power of natural killer cells and cytotoxic T cells (68).

Natural killer cells, a special kind of lymphocyte cells, are the next fighters of the innate immune response. These cells kill only those cells which lack MHC-I on their surface (69). Once they come in contact with cells, they release perforin and create pores in the plasma membrane of the invaded cells. Consequently, ions and water rush inside the infected cell leading to cell swell and burst (70).

Unfortunately, the dysregulated innate immune response is observed in COVID-19 patients. A higher level of pro-inflammatory cytokines like IL-1, IL-6, TNF-α, and chemokines 6are noted in the serum of severely infected patients (71) (**Figure 2A**). A higher number of neutrophils and a lower number of lymphocytes are also observed in the patients. Cytokines and chemokines have an essential role in the innate immune response (72). A recent clinical report of 41 patients from the Huanan sea food market reported a high level ofIL-2, IL-7, IL-10, IP-10,Granulocyte colony-stimulating factor (G-CSF), MCP-1, MIP 1-α, and TNF-α, particularly in those patients who were in the ICU (73). IL-2, a soluble form of the IL-2R a chain (sCD25), a pro-inflammatory protein, is mainly secreted from activated CD4+ andCD8+T cells and dendritic cells. The presence of IL-2 in COVID-19 patients indicate activation of the adaptive immune response (73). IL-7 serves an important function for developing double-negative CD4- and CD8-anddouble-positive CD4+ and CD8+ cells inthymocytes.IL-7 works at all stages of T cell development (15). Raised levels of IL-7showed that adaptive immune response is rapidly required in patients involved in the above study (74). IL-10 is an anti-inflammatory cytokine secreted by the regulatory T cells, macrophages, dendritic cells, Th1, and Th2 cells. Irrespective of its source, IL-10 inhibits the functions of macrophages and dendritic cells and limits the functions of Th1 and Th2 cells as well as that of natural killer cells (75). It is previously reported that IL-10 production increases dysregulated immune response as it can damage the host cells. High expression of IL-10 in COVID-19 patients could be one reason behind the delayed and weak adaptive response (76).

Elevated granulocyte colony-stimulating factor (G-CSF) is a hematopoietic growth factor indispensable for the proliferation of and differentiation in neutrophils (77). It is produced by monocytes/macrophages in response to lipopolysaccharide (LPS), TNF-α, and IFN-γ. Higher levels of G-CSF could be the main reason behind the observed neutropenia in COVID-19 patients (78). IP-10or CXCL-10 is a 10kDa protein secreted by leukocytes, neutrophils, eosinophils, monocytes, epithelial, and endothelial cells in response to IFN-γ, which acts upon the CXCR3 receptors present on the activated T cells, β-lymphocytes, natural killer cells, dendritic cells, and macrophages (79). It is reported that IL-10 plays an essential role in T cell trafficking in various infections caused by parasites like *Toxoplasma gondi* (80). An elevated level of CXCL-10/IL-10 in the patients of COVID-19 at Wuhan further evidenced the exacerbation of the innate immune response.

Monocyte chemoattractant protein-1 (MCP-1), also known as CCL-2, plays an important role in chemotactic monocytes and macrophages and has a repairing role in the damaged tissue. The said effect of MCP-1 is already reported in previous studies (81). Production of MCP-1 by monocytes involves the infection of monocytes with the virus, which then releases INF-β, which acts on other leucocytes. These leucocytes secrete some unknown soluble substance that stimulates the monocytes to secrete MCP-1 protein for chemotactic purposes. The upsurged level of MCP-1 reported in the above study showed the involvement of monocytes and macrophages at the injury site due to SARS-CoV-2 (82).

Macrophage inflammatory protein1α (MIP-1α) or CCL-3 is the next cytokine observed in patients with SARS-CoV-2. Various studies have reported that MIP-1α enhances leukocyte trafficking at the site of infection (83). The movement of the leucocytes towards the injury site further augments the inflammatory response through TNF-α, IL-1, and IL-6. MIP-1α also has an essential role in CD8+ T cells chemotactic effect. Therefore, to stop further inflammatory response, inhibition of MIP-1α becomes crucial. A study on the same has already shown reduced recruitment of neutrophils when MIP-1α was selectively inhibited by an anti-MIP-1α antibody (56).

Tumor necrosis factor-α (TNF-α) is the master regulator of inflammation. It is known that TNF-α contributes to inflammation by participating in vasodilation and edema formation, enhancing adhesion of leucocytes to the epithelium, regulating blood coagulation, inducing oxidative stress in inflammation, and finally by inducing fever (84). Augmented TNF-α in the above study further evidenced the development of strong inflammation in SARS-CoV-2 patients. Approximately all patients of COVID-19 have reported the above-stated symptoms that can be blocked by TNF-α antibody-like infliximab/adalimumab (85).

Zhou et al. (86) conducted a clinical trial on eight confirmed patients of COVID-19. They observed a heightened immune response by taking samples directly from the bronchoalveolar lavage(BAL) instead of taking blood samples. Cell composition analysis of BAL fluid of COVID-19 patients showed neutrophils, eosinophils, dendritic cells, and mast cells. Interestingly, like previous studies, raised NLR was also observed in this study, which again confirms the role of NLR in COVID-19 pathogenesis (86). They also observed pro-inflammatory cytokines and chemokine genes (IL-1B, CXCL-17, CXCL-8, and CCL-2) along with specific antiviral interferon-stimulating genes (ISGs) like IFIT and IFITM in BAL. IFIT and IFITM genes belong to the family of genes called IFITs expressed by the infected viral cell to initiate INFs synthesis in nearby healthy cells and thus play an important role in the host innate immune response (86). It is previously reported that IFIT-coded proteins interfere with the viral translation process and thus with the viral replication process (48). The raised levels of INFs in COVID-19 patients would result due to overexpression of IFIT and IFITM genes to combat viral infection in nearby healthy cells (87). They also observed an upregulated level of calgranulin genes with pleiotropic functions in inflammatory disorders (S100A8, SI00A12). Interestingly, the upregulatedIL-1RN and SOCS3 were also observed, which confirms feedback inhibition of cytokines as both these genes have an antagonistic function on cytokine synthesis. Among the upregulated cytokines, CXCL-17is observed as highly expressed in all SARS-CoV-2 patients, highlighting its role in COVID-19 pathogenesis (88). CXCL-17has a major chemoattractant role in the mucosal tissue during cellular injury, especially in the lungs. The chemotactic neutrophils further exacerbate inflammation by CXCL-8, CXCL, and CXCL-2 as these cytokines play a crucial role as neutrophil chemoattractants (89).

While most studies on SARS-CoV-2 shed light on the innate immune response, few studies also reported activation of the adaptive immune response in COVID-19 patients. A recent survey of 34 hospitalized patients evidenced the activation of humoral-mediated response (part of the adaptive immune response) in SARS-CoV-2-infected patients. The blood antibodies, IgG and IgM levels, were carefully monitored for up to four months. It was concluded that IgG antibody level continuously kept increasing after recovery in SARS-CoV-2-infected patients while the blood level of IgM first increased and then kept on decreasing. This study evidenced the activation of B cells producing specific antibodies against SARS-CoV-2antigens (90).

Another study was carried out by Eugenia Ziying Ong and colleagues, who reported a high-level expression of IL-1 in severe cases of SARS-CoV-2 (91). A similar study was conducted using the blood samples of COVID-19 patients. The severity of infection was described based on cytokines IL-6, IL-8, and IL-10 in cellular microparticles (cMPs). These cMPs were reported to contain cellular receptors, cytoplasmic proteins, nucleic acids (RNA, micro-RNA, and DNA), and cytokines. A high number of cMP was written in the blood of COVID-19 patients compared to that of healthy persons. Upon cytokine analysis, a higher level of IL-6, IL-8, and IL-10 was detected in severe pneumonia patients. Low levels of IL-6 secreted by macrophages can protect the lung alveoli. Still, the excessive release of IL-6 can adversely affect them by inducing fibrinogen activation and activation of coagulation factors, inhibit endothelial repair, and thus increase the permeability of blood vessels which causes inflammatory lung injury. IL-8, with its strong neutrophil chemotactic and activation potential, can further induce inflammation. Like IL-6, a low level of IL-8 protects the lungs, but a higher level can damage them. On the other hand, IL-10 has an anti-inflammatory action and can monitor the host immune response through T helper cells; it can also inhibit overexpression of pro-inflammatory cytokines and thus can serve as the best prognostic marker to control the host immune response along with IL-6 and IL-8 (92).

There is an increase in antibody-secreting cells (ASCs), follicular helper T cells (TFH-cells), and SARS-CoV-2-specific IgM in mildly infected patients, and IgG is observed before symptomatic recovery. Lower levels of CD16+and CD14+ subset monocytes were also reported in the same study, indicating immunopathology and recruitment of these cells at the site of action (**Figure 2B**) (3). Monocytes, a part of innate immunity, are mainly involved in phagocytosis of the infected cells, but subsets of CD16+ and CD14+ expressing monocytes also indicate their role in adaptive immunity (93).

In severely infected patients, a high level of differentiation of subsets of macrophages has been reported. RRR et al. first found four subgroups of macrophages classified as FCN1, SPP1, and FABP4 markers in severely infected patients. Among subgroups 1 and 2, macrophages are recognized as FCN1+ (monocytes-derived) macrophages. These express higher inflammatory mediators like cytokines, CCL-2, CCL-3, CCL5, IL-8, CXCL-10, and CXCL11, and hence play a major role in inflammation of the alveolar sac. Contrary to this, subgroup3 macrophages, i.e.,SPP1+ macrophages, are observed to repair function rather than damage the alveoli (94). Overall, an opposite effect of SPP1+ macrophages on FCN1+ macrophages has been observed on the alveoli. The relative concentration of the two types of macrophages would determine if there would be an inflammation response or repairing action on the alveoli. The fourth group, FABP4+ alveolar macrophages, showed higher expression of PPARγ, which plays a vital role in the metabolism of the lipid surfactant. It is previously reported that the lipid surfactant is essential for the efficient working of alveoli as it has a major role in reducing alveolar surface tension. A decrease in the productivity of the surfactant can lead to the collapse of the alveolar sac and hence respiratory failure in the infected patients. Therefore, PPARγ-expressing FABP4+ alveolar macrophages somehow enhance the synthesis and secretion of lung surfactant (95).

In severely infected patients, dysregulated innate immune systems accumulate fluid in the gap between alveolar and endothelial cells and cause difficulty breathing. Most recent studies have reported these symptoms in COVID-19 patients (96).

## Adaptive Immune Response

**Figure 3:**
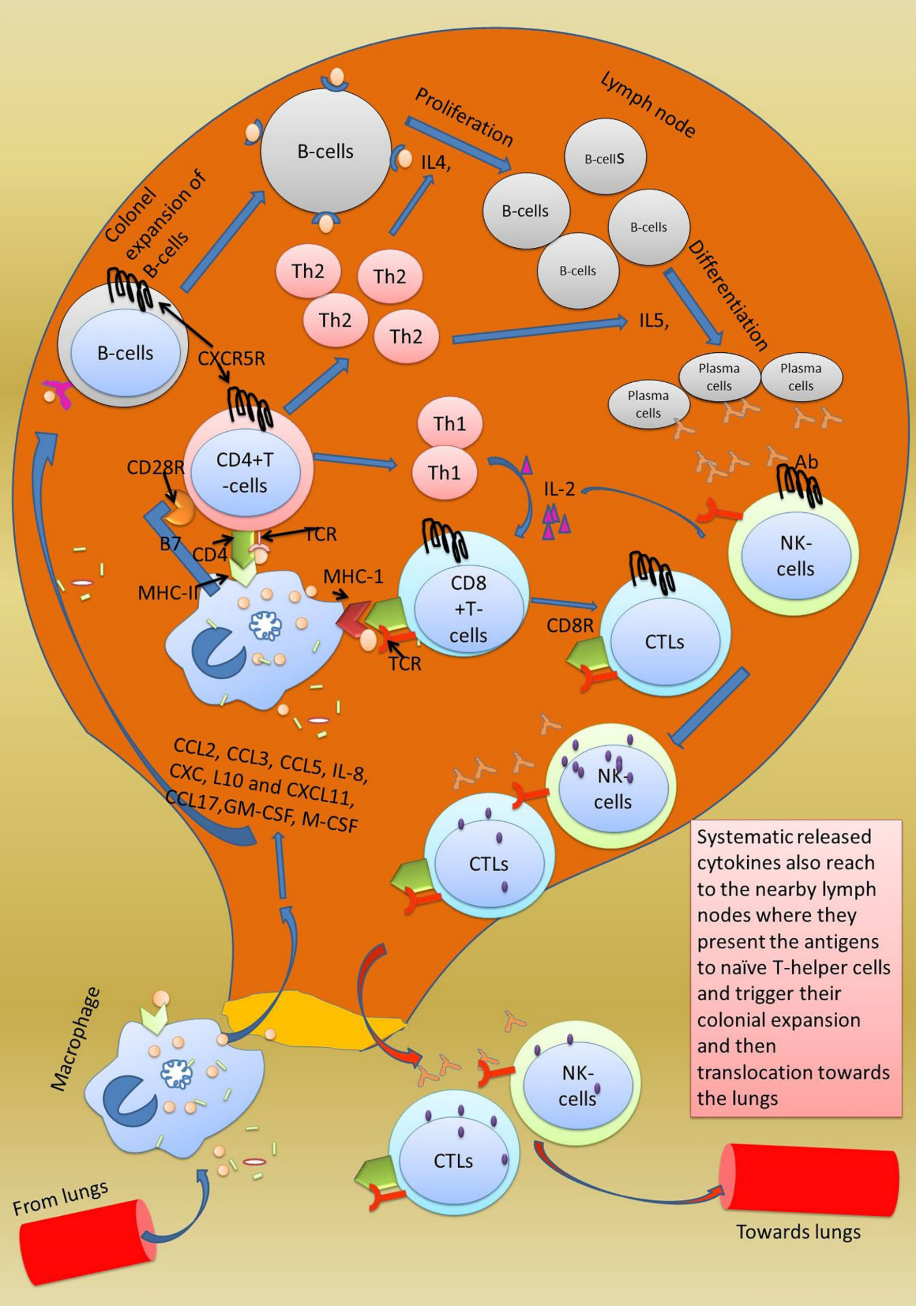
Activation of adaptive immune response in lymph nodes. Adaptive immune response is triggered when the secreted chemokines by macrophages in the lungs reach the lymph nodes and cause colonial expansion of native CD8+ T cells and B cells which rush to the site of injury and start clearing the virally infected cells. The killing of viral-infected cells damages the type-2 pneumocytes which is followed by accumulation of fluid in the space inside the alveoli. Excessive mechanical injury to the alveolar cells damages the alveolar epithelium and thus causes respiratory failure which ultimately develops into ARDS.

**Figure 4:**
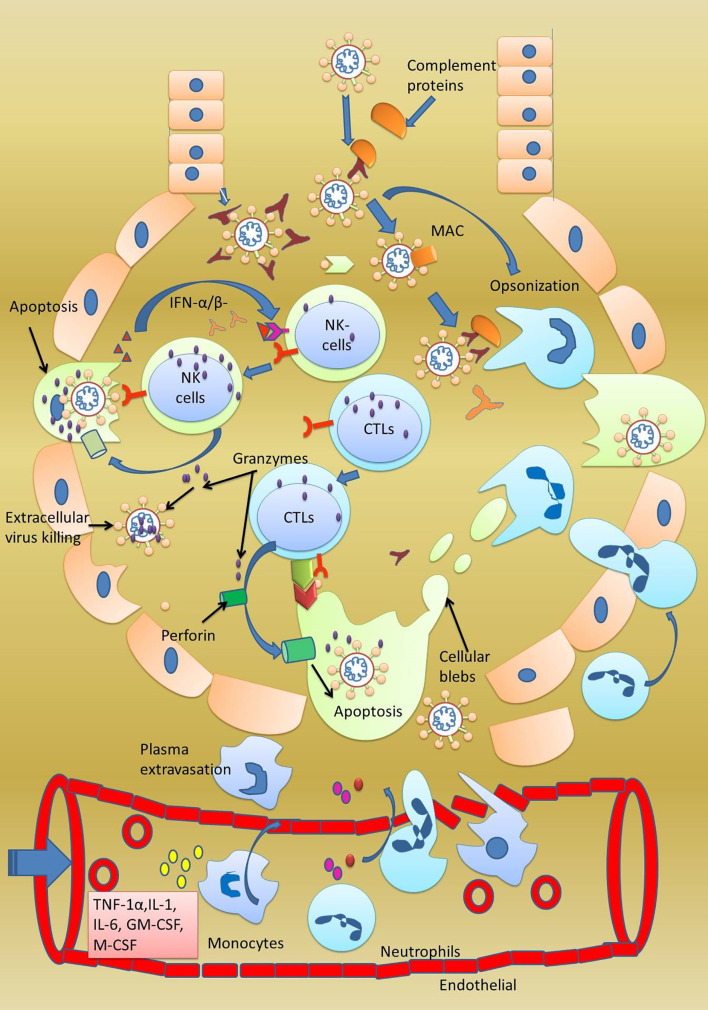
Normal adaptive immune response. Activated CTL, B cells, and natural killer cells after being activated in the lymph nodes translocate towards the lungs. In the lungs, CTL and NK cells starts clearing the SARS-CoV-2-infected cells. Both these cells kill cells by the inducing apoptosis in the target cells by secreting granzymes which form perforins in the target cells. Consequently, ions start leaking, and initiated apoptosis can be observed by formation of membrane blebs. The small membranes blebs are then phagocytosed by the neutrophils. Antibodies secreted from the B cells inhibit the virus replication by neutralization. Antibodies also help in opsonization and phagocytosis of the virus by neutrophils.

Adaptive immune response specifically kills bacterially and virally infected cells. It uses three reactions: humoral response, antibody-mediated response, and cell-mediated response, which uses specific cytotoxic cells (97). It is activated when neutrophils release phagocytized antigen fragments into the circulation and when antigen-presenting cells (macrophage, dendritic cells) reach the lymph nodes (**Figure 3** **)** (98). Lymph nodes have a particular type of cells known as CD4+T cells (differentiated into T helper cells (Th1 and Th2) and CD8+T cells [differentiated into cytotoxic T lymphocytes (CTL)] which are stimulated by the antigen-presenting cells (APC) (99). Further, the differentiation of βcells into antibody-secreting cells depends on IL-4 provided by activated T helper cells. Macrophages present their processed antigen on MHC-II molecules that bind with CD4+ T cell receptors of the T helper cells. The antigen on MHC-II is recognized by the T cell receptor (TCR). Co-stimulation of T helper cells is achieved through CD28+ receptors on T helper cells and B7 factor on macrophages (100). The final step in the activation of T helper cells involves secretion of IL-1, and it binds toIL-1 R on the T helper cells **(** **Figure 4** **)**. As a result, T helper cells get activated and undergo auto-activation with IL-2,and T helper cells 1 (Th1) proliferate into numerous T helper 2 cells (Th2),which further enhances the expression of IL-4 and IL-5. The secreted IL-4 and IL-5 serve essential functions on β cells. IL-4 enhances the colonial expansion of βcells, and IL-5 triggers their differentiation into antibody-secreting plasma cells (101). Secreted antibodies neutralize the circulating antigen and enhance the clearing of the pathogen by activating the complement system, as discussed above. Poor activation of antibodies secreting βcells has been reported in COVID-19 patients. According to the findings of one study, antibodies were produced against the RBD of the spike protein and nucleoprotein in the COVID-19 patients with a high viral load. After 20 days of hospitalization, viral RNA was continuously detected in the posterior oropharyngeal saliva of those patients, indicating poor activation of βcells (102).

Another important player of adaptive immune response is cytotoxic T lymphocytes (CTLs) or killer T cells or CD8+ T cells activated by virally infected macrophages. Circulating virally infected macrophages reach the lymph nodes where they present the processed antigen to CD8+ T cells. Macrophages interact with their MHC-II molecule with the CD8+ receptor of cytotoxic T cells. Once stimulated by the antigen, CTLs from lymph nodes start traveling towards the site of infection through the bloodstream. The antigen on the MHC-II is read by the CTLs, which in response starts producing perforin and granzymes. Perforin creates pores in the virally infected cells through which granzymes enter the same cell and initiate apoptosis of the infected cell (68).

The adaptive immune response also comes into action along with the innate immune response in severely infected patients. The adaptive immune response uses two weapons antibodies-secreting βcells and T cells (cytotoxic T lymphocytes orCD8+ T cells and CD4+ T cells) to clear the virally infected cell. CD4+ T cells act as T helper cells programmed to respond only to MHC-II-expressing cells, while CD8+ T cells are programmed to respond only to MHC-I-expressing cells. Along with these cells, natural killer cells, although a part of innate immunity, play an important role in killing SARS-CoV-2-infected cells. Firstly, all those cells that lack MHC-1 protein on their surface are killed. Secondly, all those cells with the MICA protein on their surface are killed. Thirdly, all those cells with attached IgG antibodies on their surface antigen are also killed. Natural killer cells also induce apoptosis in the target cells. Another important point is that adaptive immunity clears the viral infection and memorizes the invading pathogen, and protects us from future infections from the same pathogen (103).

**Figure 5:**
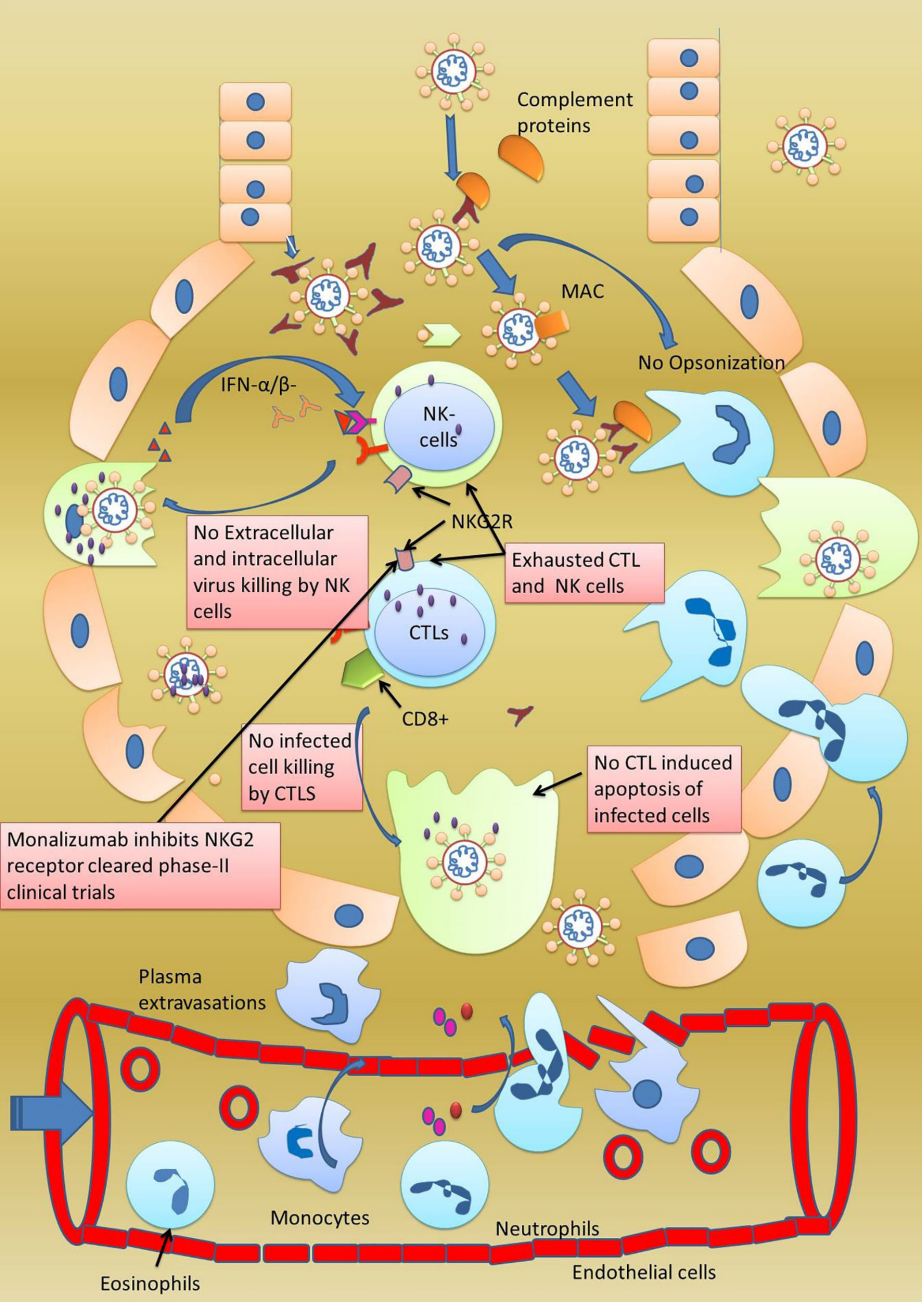
Exhaustion of adaptive immune response. In some patients of infected with SARS-CoV-2, although adaptive immune response was observed to be activated by the presence of CTL, NK, and B cells, none of these cells were found killing the virally infected cells. The special behavior shown by these cells was termed as exhaustion of NK, CTL, and B cells. Furthermore, these cells were also observed to be expressing NKG2 on their surfaces making NKG2 as important drug target. Monalizumab, a NKG2 receptor inhibitor, has been reported to clear a phase-II clinical trial.

A recent study has reported high colonial expansion of CD8+ T cells in COVID-19 patients. At the same time, abnormally delayed adaptive immune response was observed in some patients infected with COVID-19 (55). Zheng et al. stated that cytotoxic T lymphocytes and NK cells are dispensable in controlling the viral infection. They conducted a study on 68 COVID-19 patients to monitor their CTLs and NK cell levels in their blood plasma **(** **Figure 5** **). **With the progression of the disease, a continuous decrease inCD8+ cells were noted. They also reported that among lymphocyte populations, CD8+ and NK cells are involved mainly in anti-COVID response. The number of T cells and CD8+ cells was low in patients with severe disease in comparison to patients with mild disease. Patients with COVID-19 showed a functional exhaustion of NK and CD8+ cells. This exhaustion of NK and CD8+ cells showed an increase expression of the CD94/NK group 2 member A (NKG2A) receptor. Interestingly, in patients after therapy, the number of NK and CD8+ cells were restored and the expression of NKG2A was reduced. These findings allow us to hypothesize that the functional exhaustion of cytotoxic lymphocytes associated with COVID-19 infection breaks the antiviral immunity and enhanced expression of NKG2A as specifically observed in CD8+ and NK cells could contribute to the maintenance of this blunted antiviral surveillance (104). It is well known that IFN-γ, IL-2, granzyme-B, and TNF-α are responsible for the proliferation of βcells into antibody-secreting plasma cells and colonial expansion of NK cell/CTL. The inhibition of these cytokines’ expression by these cells could be a strategy used by SARS-CoV-2to suppress the adaptive immune response. The NK cells and CTL are responsible for clearing the virus-infected cells and since the virus here does not want to be removed by the NK cells and CTL, enhancing NKG2 expression on these cells helps achieve that objective. This could explain why some COVID-19 patients remain asymptomatic for a long time. Moreover, a decrease in the levels of NKG2 and the cytokines above are reported. This makes NKG2 a vital drug target in the immune checkpoint to prevent SARS-CoV-2 replication (96). Delayed or no activation of T cells is further supported by another study conducted on three positive COVID-19 patients and10 healthy persons, which concluded that delayed response is a trick used by the SARS-CoV-2 virus to prolong its infection and to maintain a febrile environment so that it can enhance community transmission (105). Abnormally low levels ofCD3+, CD4+, and CD8+ T cells were noted in severely infected COVID-19 patients. These cells are specific for viral infection, and their lower number indicates severe dysregulation in the adaptive immune response (86).

## Host Immune Response and Development of Severe Pneumonia

Lung alveoli are spaces for gaseous exchange in and out of the body. The soft lining of the alveoli consists of a single layer of type-1 and type-2 pneumocytes. Type-1 cells mainly function in gaseous exchange, but type-2pneumocytes also secrete lung surfactant in addition to gaseous exchange to reduce surface tension. Continuous secretion of the surfactant from type-2 pneumocytes prevents the lungs from collapsing. The same thing is observed with SARS-CoV-2 infection, which binds toACE-2 pneumocytes and stops surfactant secretion. Infected type-2 pneumocytes trigger the host immune response by releasing inflammatory mediators that act upon the alveoli resident macrophages (106). Activated macrophages release cytokines IL-1, IL-6, and TNF-α, which activate chemotactic immune cells circulating in the bloodstream. They also act locally on the endothelial cells causing a gap between tight junctions. Due to vascular permeability of the endothelial cells, fluid leakage in the gap between alveolar epithelial and blood vessel endothelial cells increases, and vascular fluid accumulates around the alveoli damaging the epithelial cells. Blood neutrophils are attracted at the site of the infection and release reactive oxygen species, which start destroying the alveolar epithelium. Following neutrophils, monocytes also reach the site and increase phagocytosis of damaged pneumocytes and viral particles (84). Similar results have been published in a study conducted by Shan et al., who observed the development of acute pneumonia in *Rhesus Macaques* on inoculation with SARS-CoV-2. The results were spotted with chest radiographs and histopathological results. The collective response of macrophages, neutrophils, and monocytes induces excessive production of cytokines, mucus, and antibodies, which overfill the alveoli and block the gaseous exchange, leading to the death of the patient due to respiratory failure (**Figure 4**) **(** 107).

## Conclusion

Novel coronavirus SARS-CoV-2 transmitting from person to person requires host alveolar type-2 pneumocytes to complete its life cycle. Numerous ACE-2 receptors on the apical side of the type-2 pneumocytes provide an interface for viral entry. SARS-CoV-2 brings forward its spike protein for attachment with ACE-2 receptors. Its spike protein is a trimeric protein that has a furin protease cleavage site. Intracellular serine protease TMPRSST serves this function and causes the cleavage and activation of spike proteins. Upon activation, the S1 subunit of the spike protein binds to ACE-2 receptors, and the S2 subunit initiates fusion with the plasma membrane of type-2pneumocytes. Some studies have also reported that host ADAM17 and cathepsin B/L serve the same function in failing TMPRSS2 to execute the activation of the spike protein. Once in the host cells, SARS-CoV-2uses host ribosomes to synthesize its structural proteins and genomic virions. Upon completion of its life cycle, the progeny virus bursts the cell and is ready to transmit the infection in healthy individuals (40).

The host immune system starts its function once the infection is complete. Interestingly, different responses from the host immune system have been observed in COVID-19 patients, depending on the severity of pneumonia (103).In mildly infected patients, only a small elevation in IgG and IgM antibodies and fewer cytokines are observed, which impart protection to the lungs. Moderate cases of COVID-19 have a higher level of antibody-secreting cells, macrophages, neutrophils, along with a high level of cytokines. In patients with severe pneumonia, a very high level of cytokines (IL-6, IL-8, IL-10, and TNF-α) and large subtypes of macrophages have been observed but no activation of NK cells CD8+ T cells is evident (108).

From the reported data of COVID-19 patients, it is tough to understand whether how the host immune system acts is pivotal. This is simply due to the dual behavior of the immune system. It acts as a hero within limits but turns into a villain whenever there is an excess of a particular cytokine. Another reason for this type of behavior could be the specific type of organ or tissue or cells. Since lung alveoli, the site of gaseous exchange, are made of a single layer of type-1 and type-2 pneumocytes, injury to single cells means losing a large surface area. Overactivation of inflammatory markers can overstimulate the immune system, which could damage and reduce this surface area through its phagocytotic action. This is why NK cells and T cells remain inactive in mild and moderate COVID-19 patients (105). In severe pneumonia patients, the joint function of macrophages, neutrophils, monocytes, natural killer cells, and CD8+ T cells damage and phagocytose the alveolar type-1 and type-2 pneumocytes; this means that COVID-19 patients must be placed on a ventilator. In most instances, patients die of respiratory failure (109).

It would be pertinent to mention that a physician can save more lives by controlling the patient’s immune response. One way to control the hyperactive immune response is to use anti-inflammatory drugs (95). Another way of controlling the hyperactive immune system is to stop the proliferation of inflammation, enhancing FCN+ macrophages, and accelerating the expansion of anti-inflammatory SPP+ macrophages. Something can be achieved by controlling specific cytokines, i.e., inflammatory cytokines IL-6, IL-8, TNF-αcan be neutralized with antibodies, explicitly increasing the anti-inflammatory cytokine IL-10. Excessive phagocytosis by the NK cells and CTL in severely infected patients can be managed by regulating the NKG2 receptors on these cells. Low NKG2 expression on NK cells and CTL makes them more active, while high expression makes them less involved and less phagocytic (**Figure 5**). Therefore, by working at various immune checkpoints through innovative *in vitro* disease models, a physician can reduce the mortality associated with the SARS-CoV-2 virus (6, 110).

The current review provides a detailed study of the COVID-19 viral life cycle and host immune response, which may give a new direction to researchers in developing various treatment strategies to overcome the infection caused by the novel coronavirus.

## Author Contributions

LS contributed to the generation of the hypothesis and manuscript writing. SB, MG, and NV helped create the scientific illustration. MNA and AS checked the whole manuscript for grammar and plagiarism errors. GK and MS perceived the idea, designed and supervised the whole study, and prepared and proofread the final manuscript. All authors contributed to the article and approved the submitted version.

## Conflict of Interest

The authors declare that the review was conducted in the absence of any commercial or financial relationships that could be construed as a potential conflict of interest.

## Abbreviation

ARDS, Acute respiratory distress syndrome; SARS-CoV-1, Severe acute respiratory syndrome coronavirus-1; COVID-19, Coronavirus disease 2019; MERS: Middle East respiratory syndrome; 5. SARS-CoV-2, Severe acute respiratory syndrome coronavirus-2; ACE-2 receptor, Angiotensin converting enzyme 2 receptor; RAS: Renin angiotensin system; TMPRSS2, Transmembrane serine protease 2; ADAM-17, A metalloproteinase protein belonging to the adamylysin surface protein; NK cells, Natural killer cells; IFNs: Interferons; NETS, Neutrophil extracellular traps; MAC, Membrane attack complex; MHC-I&II, Major histocompatibility complex-I and II; ILs, Interleukins; G-CSF, Granulocyte colony stimulating factor; IL-10, Interferon γ -induced protein 10kDa also known as C-X-C motif chemokine; MCP-1, Monocyte chemoattractant protein-1; TNF-α, Tumor necrosis factor-α; Th1&Th2, T helper cells type 1 and 2; MIP-1α, Macrophage inflammatory protein-1α; BAL, Bronchial alveolar lavage; NLR, Neutrophil leucocyte ratio; ISGs, Interferon stimulating genes; IFITs, Interferon-induced protein with tatratricopeptides repeats; cMPs, Cellular microparticles; ASCs, Antibody secreting cells; TFH-cells, Follicular helper T cells; PPARγ, Peroxisome proliferator activated receptor-gamma; CTLs, Cytotoxic-T lymphocytes; APC, Antigen presenting cells; RBD, Receptor binding domain; MICA, MHC class 1 polypeptide-related sequence A.
